# Is pre-operative urodynamic bladder function the true predictor of outcome of male sling for post prostatectomy incontinence?

**DOI:** 10.1007/s00345-020-03288-8

**Published:** 2020-06-06

**Authors:** Bogdan Toia, Lap Yan Leung, Raveen Saigal, Eskinder Solomon, Sachin Malde, Claire Taylor, Arun Sahai, Rizwan Hamid, Jai H. Seth, Davendra Sharma, Tamsin J. Greenwell, Jeremy L. Ockrim

**Affiliations:** 1 University College London, Gower St, Bloomsbury, London, WC1E 6BT UK; 2 Department of Urology, University College London Hospital, 16-18 Westmoreland Street, London, W1G 8PH UK; 3 St George’s University Hospital, Blackshaw Rd., Tooting, London, SW17 0QT UK; 4 Guy’s and St Thomas’ NHS Foundation Trust, King’s College London, London, SE1 9RT UK

**Keywords:** Urodynamic, Men, Post prostatectomy, Stress urinary incontinence, Male sling

## Abstract

**Purpose:**

To investigate pre-operative urodynamic parameters in male sling patients to ascertain whether this might better predict surgical outcomes and facilitate patient selection.

**Methods:**

We performed a retrospective, case notes and video-urodynamics, review of men who underwent AdVanceXP male sling in three London hospitals between 2012 and 2019. Urodynamics were performed in all centres, while retrograde leak point pressure (RLPP) was performed in one centre.

**Results:**

Successful outcome was seen in 99/130 (76%) of men who required one pad or less per day. The dry rate was 51%. Pad usage was linked to worse surgical outcomes, mean 2.6 (range 1–6.5) for success vs 3.6 (range 1–10) although the ranges were wide (*p* = 0.002). 24 h pad weight also reached statistical significance (*p* = 0.05), with a mean of 181 g for success group versus 475 g for the non-successful group. The incidence of DO in the non-successful group was significantly higher than in successful group (55% versus 29%, *p* = 0.0009). Bladder capacity less than 250 ml was also associated with worse outcomes (*p* = 0.003). Reduced compliance was not correlated with outcomes (31% for success groups vs 45% for non-successful group, *p* = 0.15). Preoperative RLPP was performed in 60/130 patients but did not independently reach statistical significance (*p* = 0.25).

**Conclusion:**

Urodynamic parameters related to bladder function—detrusor overactivity and reduced maximum cystometric capacity predict male sling outcomes and may help in patient selection for male sling (or sphincter) surgery; whereas urodynamic parameters of sphincter incompetency (RLPP) were not predictive. Further larger scale studies are required to confirm these findings.

## Introduction

The number of radical prostatectomies has more than doubled over the last 20 years, with 9844 performed in the United Kingdom in 2018–2019 [1]. Most prostatectomies are now performed using robot-assisted laparoscopic techniques. The incidence of post-prostatectomy incontinence (PPI) in the robotic era has been reported as 5–20% at 12 months [2, 3]. This equates to an estimated 300–1300 new patients with persistent PPI every year.

Current treatment options for men who fail conservative therapy include the Artificial Urinary Sphincter (AUS; AMS 800, Boston Scientific) which is currently considered the gold standard, or the male sling (Advance XP, Boston Scientific) [4]. The AUS is effective in an estimated 80% of men [5] but requires manual dexterity and cognition to operate [6] and long-term data suggest risk of malfunction in up to 23–25% and erosion in 4–7% [7, 8]. Male slings do not contain mechanical elements, and appeal to patients who do not wish to manipulate a scrotal pump and avoid the risks of long-term malfunction. Despite their theoretical appeal the published data on male slings report wide variations in outcome [9]. The reasons for such variation are not entirely clear, but are presumed to be in part due to the selection criteria for intervention [10, 11]. Identifying the patient characteristics that could predict surgical outcome is of vital importance in improving patient selection and consent.

To date, little data have been published on the role of pre-operative urodynamic parameters in predicting surgical outcomes of male slings. Urodynamic testing in patients with a history indicative of stress incontinence or a positive cough test is not universally recommended by clinicians and guidelines (American Urological Association and the Society of Urodynamics, Female Pelvic Medicine and Urogenital Reconstruction) [12]. Other clinicians believe that urodynamic studies provide important information on multiple facets of sphincter and bladder function [13]. These parameters may guide patient counselling for surgery when deciding between sling implant or artificial sphincter.

We, therefore, conducted a retrospective review of urodynamic data prior to male sling surgery and correlated this with outcomes from three London teaching hospitals.

## Patients and methods

AdVanceXP™ male slings were introduced into clinical practise in three tertiary Urology centres in London in 2012. All men were offered a male sling as alternative to an artificial urinary sphincter after at least 12 months of conservative therapy, which included pelvic floor exercises and medical treatment for overactive bladder. We retrospectively reviewed data including surgical history, urodynamic findings, retrograde leak point pressure (RLPP) where available, complications, functional outcomes and the need for further surgery.

Urodynamic investigations were performed after failure of 12 months conservative therapy. RLPP was performed immediately before video-urodynamic testing in one of the three centres. The technique utilised was described by Comiter et al. [14] and included the modifications suggested by Solomon et al. [15]. Subsequently video-urodynamics were performed utilising a 6F dual lumen transurethral catheter at all institutions according to ICS protocols [16]. The (video) urodynamic platforms utilised were Laborie (in 2 centres) and Genesis for the initial cases up to 2017, followed by Laborie in the third centre. Parameters assessed included presence of DO, compliance loss, maximum cystometric capacity (MCC) and RLPP. Reduced compliance was defined as increase of intravesical pressure of over 1 cmH_2_O for each 40 ml of solution infused [17].

Male sling implantation was performed by standardised technique at all three institutions as defined by criteria from the MASTER study protocol [18].

Outcomes were defined using number of pads and categorised as successful (one pad or less for reassurance) or failed (two or more pad use). Patients who initially required one per day were only considered a success if they were completely dry following surgery. Patients were followed up at 3 months, 12 months and yearly thereafter.

Statistical analysis was performed using independent t test and Chi-squared as appropriate utilising IBM SPSS Statistics 22. A *p* value < 0.05 was considered statistically significant.

## Results

From 2012–2019, 134 men underwent AdVanceXP™ Male Sling insertion for post-prostatectomy incontinence across the three centres, with complete pre-operative urodynamic data available for review. We excluded four patients with irretrievable urodynamic data.

The median patient age was 67.2 years (range 47–88). Previous prostate surgery was radical retropubic prostatectomy in 128 men (robot assisted *n* = 96, laparoscopic *n* = 19, open abdominal (*n* = 12) or perineal (*n* = 1) approach. Two patients with benign disease (transurethral resection of the prostate or HoLEP) were also treated with male sling and are included in the dataset. The mean interval between prostate surgery and male sling insertion was 48.2 months (range 11–270). 17 men had radiotherapy either prior to the sling insertion or immediately (within 3 months) after surgery. Mean follow-up is 25.5 months (range 6–72). Outcomes represent those from the latest clinic appointment.

99/130 (76%) of men required one pad or less per day, of which 66 men (51%) of men did not require any pads (dry). Only 31 (24%) men of patients had significant residual leakage at the last follow-up.

**Fig. 1:**
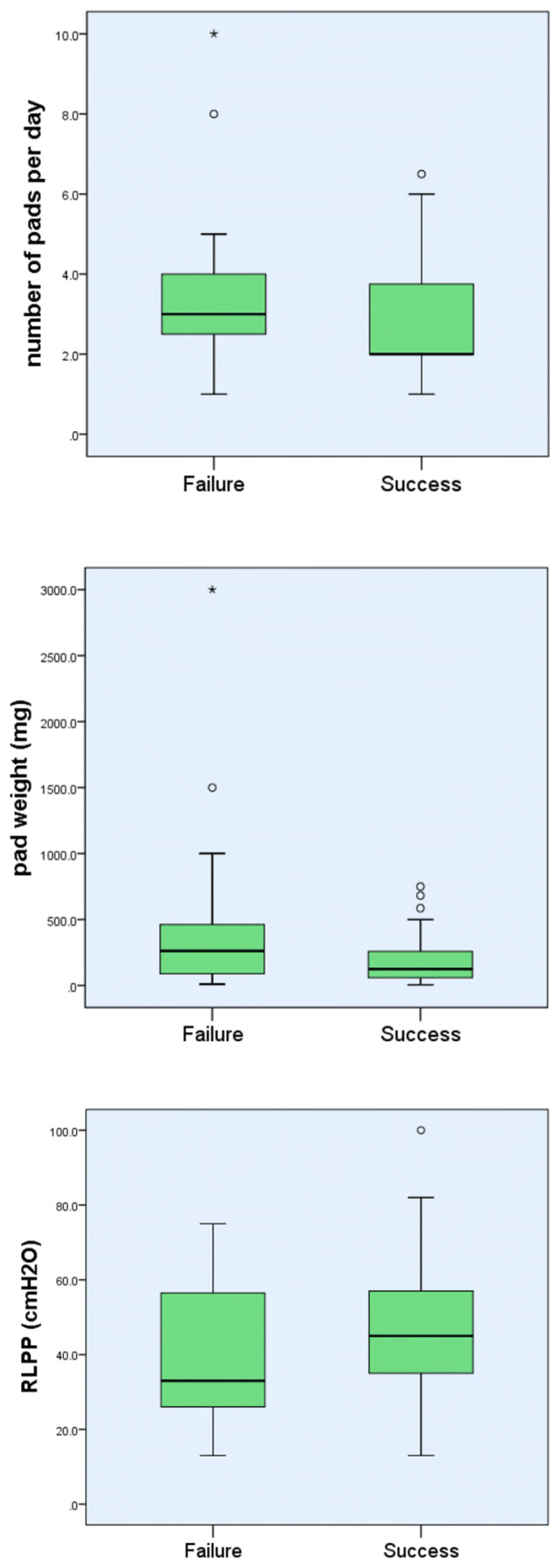
Pad usage (number of pads), pad weight (grams) and RLPP (cmH_2_O) as predictors of surgical outcome. Mean (bars) and ranges (whiskers) shown

Pre-operative incontinence was quantified using number of pads per day (mean 2.87, range 1–10). Two men relied totally on convenes, while 17 men required only one pad per day. 24-h pad weights were recorded in 90 men (mean 253 ml, range 5–3000 ml). The number of pads preoperatively was linked to worse surgical outcomes [mean 2.6 (range 1–6.5) for success vs 3.6 (range 1–10) for the non-successful group, *p* = 0.002] although the ranges were wide. The 24 h pad weight also reached statistical significance (*p* = 0.05), with a mean of 181 g (range 5–750 g) for success group versus 475 g (range 10–3000 g) for the non-successful group (Fig. 1). Using the commonly used threshold of 400 g success rate was achieved in 9/18 (50%) patients, whilst pad weight less than 400 g resulted in success rate in 59/72 (81.9%) patients (*p* = 0.005).

**Fig. 2:**
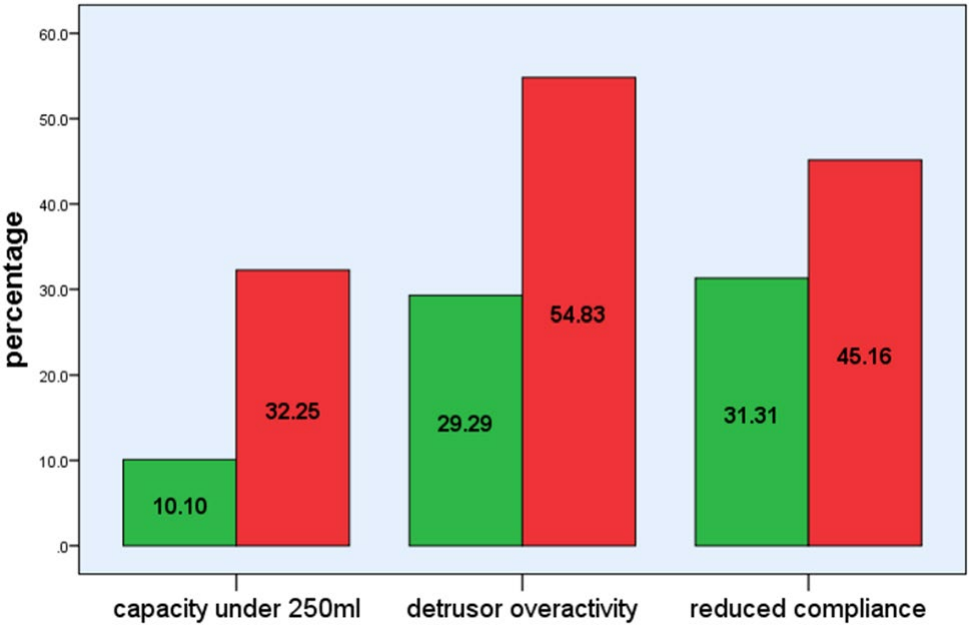
Frequency of preoperative urodynamic parameters grouped by surgical outcome

Success by urodynamic parameters is shown in Fig. 2. Time since prostatectomy and type of prostatectomy did not influence outcomes (*p* = 0.68, 0.95). Four patients had worsening incontinence following surgery, as defined by increased pad usage, and are categorised as failures.

46/130 (35%) had detrusor overactivity (DO) on pre-operative urodynamics, with a mean pressure of 41.6 cmH_2_O (range 15–78 cmH_2_O). The incidence of DO demonstrated in the non-successful group was significantly higher than in the success group (55% versus 29%, *p* = 0.009), but the peak DO pressure did not correlate with outcomes (*p* = 0.63). 45/130 (34.6%) had abnormal compliance (increase of intravesical pressure of over 1 cmH_2_O for each 40 ml of solution infused), but compliance loss did not reach statistical significance between the two groups (45% vs. 31%, *p* = 0.15). The success group had a larger mean capacity (413 ml vs 337 ml, *p* = 0.004). 20 (15%) patients had a bladder capacity of less than 250 ml and this was associated with worse postoperative outcomes (*p* = 0.003).

RLPP was performed in 60/130 patients but did not independently reach statistical significance (*p* = 0.25) as a predictor of surgical outcome. Radiotherapy was not associated with poorer outcomes (*p* = 0.32).

24 (18%) of men experienced postoperative complications. 16 (12%) had temporary urinary retention but all had resolved at last follow-up. Voiding dysfunction postoperatively was suggestive of increased chance of continence although this did not reach statistical significance (*p* = 0.07). Eight men experienced prolonged postoperative pain for up to 10 months and two men had scrotal haematomas (with associated wound infection in one man) that were managed medically.

43 (33%) men had symptoms of overactive bladder postoperatively, of which 25 of had DO on pre-operative urodynamics (*p* = 0.001). Nine patients went on to have intradetrusor onabotulinumtoxin A injections for medically refractory symptoms; two declined second line treatments.

## Discussion

Urinary incontinence is widely recognised as one of the most debilitating complications following prostatectomy, with significant impact of cancer survivors’ quality of life. Recent research on the psychological burden of PPI has demonstrated the extent of its detrimental effects, including increased cognitive dysfunction, anxiety and depression [19, 20]. As a consequence, the selection of surgical treatment for PPI, and predicting the outcomes of surgery are critical metrics for prostate cancer survivors.

The male sling is an attractive alternative to an AUS avoiding the implantation of a mechanical device with a scrotal pump, and the risks of device malfunction. Although there are several male slings on the market, the current ethos in the UK (following mesh issues in women) is that male slings should only be used within a study setting. The Advance XP (Boston Scientific) is the sling used by most UK clinicians, and selected for use in the multicentre UK MASTERs study [18]. Indeed, utilisation of multiple slings would have led to complex discussion related to heterogeneity and direct comparability of outcomes. The published outcomes for (Advance) male sling implants vary from 33–94% [11]. Most authors have focused on pad usage, particularly pad number as the primary metric for suitability (alternative to artificial sphincter) [10, 11], but little data exist on the utility of urodynamic study in identifying patients who are most suitable for a sling implant.

A widely held belief amongst clinicians is that AUS are more suitable for severe stress urinary incontinence, whereas mild incontinence would be amenable to a male sling procedure. Population characteristics (range of incontinence) in our cohort were similar to those of previously published studies [21–25]**.** Many authors have suggested a threshold of 400 mg as defining moderate from severe incontinence [21, 22], but there are little data to support this theory or this particular threshold [23].The grading of incontinence severity is usually done with number of pads or a (24 h) pad test [24]. Our data confirm better outcomes after male sling insertion for mild incontinence, with the pre-operative number of pads being a predictor of success (*p* = 0.002). However, the range was wide (1–6.5 success versus 1–10 failure). Patients that required 1 pad/day preoperatively had 88% success rates (14/17 men), as opposed to 63% (12/19 men) for men that used a convene or at least 5 pads/day. The 24 h pad weight also predicted outcome with a large difference in mean pad weights for success and failure [181 g (range 5–750 g) versus 475 g (range 10–3000 g)]. Unfortunately, the numbers were insufficient to create a predictive (logistical) analysis.

The main predictors of outcome were related to urodynamic parameters of bladder function. Reduced maximum cystometric capacity less than 250 ml and the presence of detrusor overactivity (but not peak pressure) were strongly correlated with outcome. We have reported similar correlations of DO (but not capacity) with artificial urinary sphincter implant [25]. This suggests that storage bladder function is critical to the success of both artificial urinary sphincter and the male sling and must be considered in pre-operative assessments. While the data set is not large enough for a meaningful logistic regression, it is interesting to observe that for the 10 men with DO, loss of compliance and small capacity success rate was only 20% whereas the 90 men with no DO, no loss of compliance and capacity over 250mls the success rate was 80.8%.

It is possible that pre-operative bladder function may be the most important predictors of differential outcome between these two options. Patients who are unable to fill and cycle their bladder to maintain capacity may be those at greatest risk of sling failure [26], although duration of incontinence (time from primary surgery) and poor compliance did not directly impact on outcomes. Bladder cycling using penile clamps is sometimes employed to improve bladder capacity prior to intervention, and sometimes employed as part of the urodynamic process to better assess anatomic bladder capacity (rather than a reflection of the severity of the leakage), but to our knowledge no data have been published. Comparative data are currently being collected, and sub analysis of the urodynamic data from the MASTERs trial [18] may further answer this question.

The perineal pressure manoeuvre—repositioning test—is a subjective assessment of sphincter coaptation and mobility proposed by Bauer and Gozzi [27]. The technique has not been standardised or independently validated; and it is not currently a mandated part of PPI assessment or routinely performed in the UK. Retrograde leak point pressure is an appealing alternative method of quantifying the severity of sphincter weakness as an alternative to pad weight and number of pads, which are influenced by factors such as levels of physical activity, fluid restriction and patient tolerance of non-successful pads (number of changes/day). Moreover, pad number (weight) may represent an indirect measurement of overall bladder function rather than sphincter function (weakness), given the impact of low bladder capacity and DO on surgical outcomes. Retrograde perfusion (RLPP) eliminates the storage bladder function and as result is a more direct measurement of residual sphincteric function [15]. RLPP was performed at one of the three institutions (in 60/130 men). Although we present the largest series of post-prostatectomy RLPPs in the published literature, the test failed to independently reach statistical significance (*p* = 0.25) as a predictor of surgical outcome. It is unclear whether is due to insufficient numbers, or whether this may reflect the fact that bladder function (capacity and DO) is more important than residual sphincter function. More work is required to assess RLPP and other measures of sphincter function in predicting outcomes.

In our cohort, only 17 patients had pelvic radiotherapy. Radiotherapy was not associated with poorer outcomes (success was 65% in the radiotherapy group versus 78% in men without radiotherapy, *p* = 0.32) in contrast to the current literature [28] However, practise changed through this time period with institutional policies to exclude irradiated patients from male sling implants following the initial cohort. The numbers are, therefore, insufficient to demonstrate statistical differences. It is unclear whether radiotherapy effect on the bulbar urethra leads to poorer reported outcome (poorer sphincter coaptation) or radiotherapy effects on the bladder function (i.e. reduction of capacity, compliance and detrusor overactivity) that cause poor outcomes. A smaller bladder capacity and the presence or absence of detrusor overactivity on pre-operative video-urodynamic testing was linked to poorer surgical outcomes in our cohort (*p* = 0.004 and *p* = 0.009, respectively) and this may suggest that storage bladder function is the predominant issue. If this is the case, then patients with radiotherapy treatment who maintain good (urodynamic) bladder function may still be candidates for male sling implants.

An important factor in opting for male slings over artificial urinary sphincters is the presumption that failed sling surgery do not complicate subsequent artificial urinary sphincter insertion. Lentz et al. reported AUS revision in 6.9% of men after a failed sling procedures [29]. In our cohort erosion occurred in two patients (18%). We believe that the best opportunity for success lies with the first intervention, and second interventions in scarred operative fields are associated with higher failure and complications rates. Patients should be counselled accordingly.

This study has limitations. It is a retrospective analysis of data; and incomplete urodynamic records prevented analyses in 4 (3%) of patients. Outcomes are based on pad usage, although patients were objectively much better, patient-reported outcome data would have been helpful in comparing objective and subjective outcomes. Future, prospective study will include such data.

## Conclusions

The Advance male sling was successful treatment in 76% of men with post prostatectomy incontinence. Predictors of significance were number and pad weight although the ranges were wide and their utility to predict outcome limited. Pre-operative reduction in urodynamic capacity and the presence of detrusor overactivity both predicted poor outcome, whereas RLPP was not predictive. Bladder function seems to be a critical factor in predicting the likely of male sling outcome. Patients should be carefully counselled on urodynamic parameters before deciding on surgical intervention.

## Data Availability

Anonymized data are available and are subject to individual institutional regulation.
